# Assessing Fishers' Support of Striped Bass Management Strategies

**DOI:** 10.1371/journal.pone.0136412

**Published:** 2015-08-25

**Authors:** Robert D. Murphy, Steven B. Scyphers, Jonathan H. Grabowski

**Affiliations:** Department of Marine and Environmental Science, Marine Science Center, Northeastern University, Nahant, Massachusetts, United States of America; Institut Maurice-Lamontagne, CANADA

## Abstract

Incorporating the perspectives and insights of stakeholders is an essential component of ecosystem-based fisheries management, such that policy strategies should account for the diverse interests of various groups of anglers to enhance their efficacy. Here we assessed fishing stakeholders’ perceptions on the management of Atlantic striped bass (*Morone saxatilis*) and receptiveness to potential future regulations using an online survey of recreational and commercial fishers in Massachusetts and Connecticut (USA). Our results indicate that most fishers harbored adequate to positive perceptions of current striped bass management policies when asked to grade their state’s management regime. Yet, subtle differences in perceptions existed between recreational and commercial fishers, as well as across individuals with differing levels of fishing experience, resource dependency, and tournament participation. Recreational fishers in both states were generally supportive or neutral towards potential management actions including slot limits (71%) and mandated circle hooks to reduce mortality of released fish (74%), but less supportive of reduced recreational bag limits (51%). Although commercial anglers were typically less supportive of management changes than their recreational counterparts, the majority were still supportive of slot limits (54%) and mandated use of circle hooks (56%). Our study suggests that both recreational and commercial fishers are generally supportive of additional management strategies aimed at sustaining healthy striped bass populations and agree on a variety of strategies. However, both stakeholder groups were less supportive of harvest reductions, which is the most direct measure of reducing mortality available to fisheries managers. By revealing factors that influence stakeholders’ support or willingness to comply with management strategies, studies such as ours can help managers identify potential stakeholder support for or conflicts that may result from regulation changes.

## Introduction

Successful management of marine fisheries hinges upon understanding and promoting rule compliance and sustainable fishing behaviors across diverse stakeholder groups often with competing interests [1–2]. Developing and implementing well-supported management strategies that account for these interests can prove to be difficult as commercial anglers, recreational anglers, and charter boat captains often compete to maintain their share of catch within a fishery. Even within stakeholder groups, fisher behavior and thus, fishing pressure, can be influenced by a wide range of social and economic factors including perceptions, motivations, social norms, and resource dependency [3–5]. Therefore, effectively managing fish populations requires implementing management strategies that promote biological productivity and also account for these dynamic relationships between the fishery and stakeholders.

While the impacts of commercial fishing on fish population dynamics has received substantial scientific and public attention, recreational and subsistence fishing has been increasingly recognized to also strongly influence fish populations [6–7]. Recreational fishers represent a highly diverse group of stakeholders and recreational fishing can significantly influence the welfare of fishing communities as well as contribute substantially to local and national economies [8–9]. For example, the direct expenditures from the striped bass recreational fishery in Massachusetts alone have been estimated at over US$600 million [9]. Additionally, recreational fishing often has strong cultural significance, such as in the tribal Pacific lamprey fishery [10] and Pacific salmon fishery [11]. Thus, the value of both recreational and commercial fishing is substantial, such that the interests of both stakeholder groups should be considered in the management process. Successful management strategies hinge upon stakeholder support and compliance, and for many fisheries this must involve both recreational and commercial fishery participants. Our study focuses on an iconic and controversial fishery in the northeast U.S. and aims to understand the perspectives of recreational and commercial fishers on the effectiveness of current management efforts and predict the degree to which they support different proposed management strategies.

Striped bass (*Morone saxatilis*) are of high economic value in the United States and are targeted heavily throughout New England and the Mid-Atlantic [12]. Vulnerable to heavy fishing pressure because of their close proximity to shorelines, striped bass catches along the U.S. Atlantic coast reached historical highs in the early 1970’s, but soon after collapsed [13]. Upon establishment of the *Striped Bass Conservation Act* in 1984, coastal states began implementing moratoriums [14], which lasted until the mid-1990’s when stocks were deemed fully recovered [15].

Currently, the recreational fishery alone is comprised of more than 3 million anglers and accounts for landings estimated at roughly 1.5 million fish per year [16–17]. While recreational harvest occurs in all states throughout their range, only seven states currently permit commercial harvest (Massachusetts, Delaware, Rhode Island, Maryland, New York, North Carolina, and Virginia), which accounted for approximately 840 thousand fish in 2012 [17]. Striped bass commercial and recreational fisheries along the Atlantic Coast are currently regulated by a complex of management regimes. An interstate management body, the Atlantic States Marine Fisheries Commission (ASMFC), decides upon management strategies using guidelines outlined in Amendment 6 of the Interstate Fishery Management Plan for Atlantic Striped Bass [18]. Through this plan, specific emphasis is given to the status of the female spawning stock biomass (i.e., % of SSB_MSY_), fishing mortality (F), and striped bass age structure. Each coastal state must enforce the required regulations set by the ASMFC or implement alternatives with equivalent standards and biological reference points. This management structure is composed of a variety of layers, one of which includes an advisory panel consisting of commercial and recreational fishery stakeholders. While this is certainly beneficial, our study would potentially allow for a larger, representative population of anglers to be considered in the management process.

Our study explores the perspectives of striped bass recreational anglers, commercial anglers, and charter boat captains/guides across two contrasting states: Massachusetts (MA), where both recreational and commercial harvesting occur, and Connecticut (CT), where only recreational fishing is permitted. While CT maintains no commercial fishery, MA commercially harvested roughly 66 thousand fish in 2012, or 8% of the national harvest [17]. CT and MA recreationally harvested 65 and 378 thousand striped bass in 2012, respectively. We conducted an online survey of licensed MA and CT anglers and assessed: 1) fisher perceptions of current management regimes 2) fisher receptiveness towards policy changes and 3) the perceived effectiveness of these potential policy changes for the health of both striped bass populations and the fisheries. For the purposes of our study, health is defined as the status (i.e. abundance and condition) of the striped bass stock, while the fishery encompasses both the stock and stakeholders involved in harvest. The concept of ‘health’ was chosen because it is a central tenet of the Magnuson-Stevens Fisheries Conservation and Management Act [19]. Our survey identified management strategies that anglers from both states perceive as effective and would be most receptive towards. Additionally, our analyses revealed several key predictors of fishers’ perceptions of fisheries management.

## Methods

To compare the perspectives of striped bass anglers from contrasting management regimes, fishers were surveyed from MA and CT. While both states contain substantial recreational fisheries, only MA permits commercial harvest. At the time of the survey, both states limited recreational fishers to two fish per day that can be no shorter than 28” (total length). MA commercial anglers were permitted to fish four days of the week during the striped bass season, in which they could harvest 30 fish per day (34” minimum size limit), with the exception of Sunday, where a 5 fish per day maximum was enforced.

Fishing licensee information was obtained from the MA Division of Marine Fisheries and the CT Marine Fisheries Division and consisted of commercial and recreational saltwater fishing license holders from 2013. In total, we compiled roughly 3,900 commercial fishers plus 155,000 and 35,000 recreational fishers from MA and CT, respectively. We randomly sub-sampled a total of 2,000 recreational fishers from each state and 1,000 commercial fishers. Sampling rates were chosen to achieve a representative sample of the population of each type of fisher in Massachusetts and Connecticut [20]. We assumed that response rates for recreational fishers would likely be ~10–20% [21], which would provide us with an adequate sample size to test whether the attitudes and perceptions of these fishers differ between these two states. Given that we expected potentially higher response rates of greater than 25% for commercial stakeholders [22], a lower sample size was chosen. Participants were sent emails and asked to participate in an online survey approximately 15 minutes in length using Qualtrics Survey Software Research Suite. All survey methods, including written consent statements, were approved by Northeastern University’s Institutional Review Board (IRB #13-11-25). Ten $25 gift certificates towards one of two outdoor stores were raffled as an incentive. The online survey was open for one month from February 7^th^ until March 7^th^, 2014, and throughout its duration, brief reminder emails were sent weekly to promote responses.

The survey can be parsed into three categories based on question type: Fisher classification, Management perceptions, and Demographic questions (Table 1). The fisher classification section of the survey documented fisher type (i.e., commercial, recreational, charter boat captains/guides), fisher state of residence, primary fishing location (i.e., state), effort allocated towards striped bass, percent of fishing effort from shore, fishing experience, fishing club membership, and tournament participation, and screened out anglers that do not target striped bass. For commercial fishers, this section also measured percent contribution of striped bass harvest towards personal and household income. The management perceptions section of the survey consisted of questions measuring fishers’ perspectives and receptiveness towards several hypothetical management changes including: reduced recreational daily bag limit from two fish per day down to one fish per day (this question was only given to recreational anglers), mandated use of circle hooks, a slot limit for the release of fish larger than a maximum length (example; 40” maximum size limit), and reduction in commercial yearly quota (only displayed to commercial anglers). These hypothetical policies were chosen for this study because they have either been utilized in other marine fisheries [23] and / or have been repeatedly identified as points of interest (either negative or positive) by recreational and commercial anglers with which we have had personal communications. Among the four potential management changes, fishers ranked their support on a scale from “strongly support” to “strongly oppose.” Supportive and neutral responses were grouped together as to identify fishers who would potentially exhibit no resistance (i.e., high compliance) to the proposed management alterations. We used a split-sample design that asked participants to consider each of the four management changes and provide their perceptions on how beneficial each would be for either the health of striped bass populations or the sustainability of the fishery. A split-sample design was used to determine if anglers perceive a disconnect between the health of the fish population and fishery. This design was chosen to examine angler perceptions of the health of the fish population versus the fishery independently of one another as to remove potential biases associated with answering both questions in a particular order (i.e., order bias) [24]. Additionally, we quantified percent circle hook usage among striped bass anglers. Respondents were also asked about their supportiveness for a maximum size limit. To identify if a threshold in support for a maximum size limit exists, respondents were presented a randomly assigned length between 36” and 44”. Another question asked fishers to grade their state’s management regime on an “*A+ to F*” scale. Lastly, the survey included basic demographic questions to record age, gender, ZIP code, occupation, education, and income.

**Table 1:** Summary of survey questions.

Question categories	
Fisher classification	Fisher type
	State of residence
	Fishing location (state)
	Percent effort towards striped bass
	Years fishing for striped bass
	Percent of striped bass fishing from shore
	Fishing club membership
	Striped bass tournament participation
	Income from commercial harvest of striped bass
Management perceptions	Effectiveness of current management
	Effectiveness of policy strategies
	Receptiveness to policy strategies
	Current circle hook usage
	Opinion of an upper size limit for recreational striped bass harvest
Demographics	Year of birth
	Gender
	ZIP code
	Primary occupation
	Highest level of education
	Total household income

### Statistical analyses

Pearson chi-squared tests were used to evaluate categorical variables (Table 2). Thus, Pearson chi-squared tests examined potential differences in receptiveness towards policy changes between “fisher type” and “state,” and the perceived effectiveness of various slot limit lengths. Statistical comparisons of circle hook usage by “fisher type” were completed using Kruskal-Wallis tests. Kruskal-Wallis tests were also used to evaluate fisher perceptions on the effectiveness of management changes towards the health of striped bass populations versus the sustainability of the fishery (α < 0.05) (Table 2). Kruskal-Wallis tests were used for the above analyses due to non-normal distributions. To identify predictors of fisher receptiveness towards the four potential management changes and fisher management grades, we applied the partition method from JMP 10.0.2. The partition method allows for the construction of classification trees that evaluate the explanatory power of assigned variables. Using *LogWorth* values, this method hierarchically identifies the strongest predictor at the top of the classification tree, while subsequent splits explain variation in the preceding variable. Only significant splits were shown in our classification trees (*P* ≤ 0.05). For all classification trees, the following factors were included in the analysis when applicable; “fisher type”, “state”, “percent effort dedicated to striped bass fishing”, “striped bass fishing experience”, “salary”, “percent personal income from the commercial harvest of striped bass”, “participation in at least one striped bass tournament per year” (binary), “membership in a fishing club or organization” (binary) and “gender.” Lastly, median grades were calculated for the fisher management grade question.

**Table 2:** Investigated questions and statistics used.

Question	Statistical Test
Do fishers’ perceptions of current management regimes vary according to some underlying variable(s)?	Classification tree analysis
Does fisher receptiveness vary among different types of fishers and among fishers in different states?	Pearson chi-squared test
Does fisher receptiveness vary according to some underlying variable(s)?	Classification tree analysis
Do fishers perceive that different slot limit maximum lengths have altered levels of effectiveness?	Pearson chi-squared test
Does circle hook usage vary among fisher types?	Kruskal-Wallis test
Do anglers perceive that policy changes will be similarly effective at promoting the health of striped bass populations and the sustainability of the striped bass fishery?	Kruskal-Wallis test

## Results

### Descriptives and Demographics

A total of 1,025 anglers completed our online survey (overall response rate: 20.5%) with 835 participants who fish in MA and 190 from CT (Table 3). Response rates provide confidence intervals between ±4–7% for all groups surveyed at a confidence level of 95% when extrapolating our results to the entire group of license holders in each state. Only 23 participants did not fish for striped bass and were consequently eliminated from the survey. Also, any comparison between MA and CT excluded commercial anglers as only the former state permits commercial harvesting.

**Table 3:** Summary of demographics and other fishing variables by state.

	Massachusetts	Connecticut
Sample Size	835	190
Gender		
Male	97%	96%
Female	3%	4%
Age–Mode	1955–1959	1955–1959
Annual income		
Under $40k	14%	8%
$40k-$60k	12%	12%
$60k-$80k	14%	19%
$80k-$100k	16%	14%
$100k-$150k	23%	23%
$150k-$200k	10%	13%
$200k-$250k	3%	4%
Over $250k	8%	7%
Type of fisher		
Recreational	59%	97%
Commercial	38%	n/a
Charter/Guide	4%	3%
Effort allocated towards striped bass fishing (%)–Mean	64%	54%
Fishing experience (years)–Mean	26.1	20.8
Effort from shore (%)–Mean	42%	49%
Member of fishing club		
Yes	24%	18%
No	76%	82%
Striped bass tournament participation		
Yes	25%	6%
No	75%	94%
Annual income from commercial striped bass harvest–Mean	10%	n/a

### Management Grade Analysis

**Fig 1:**
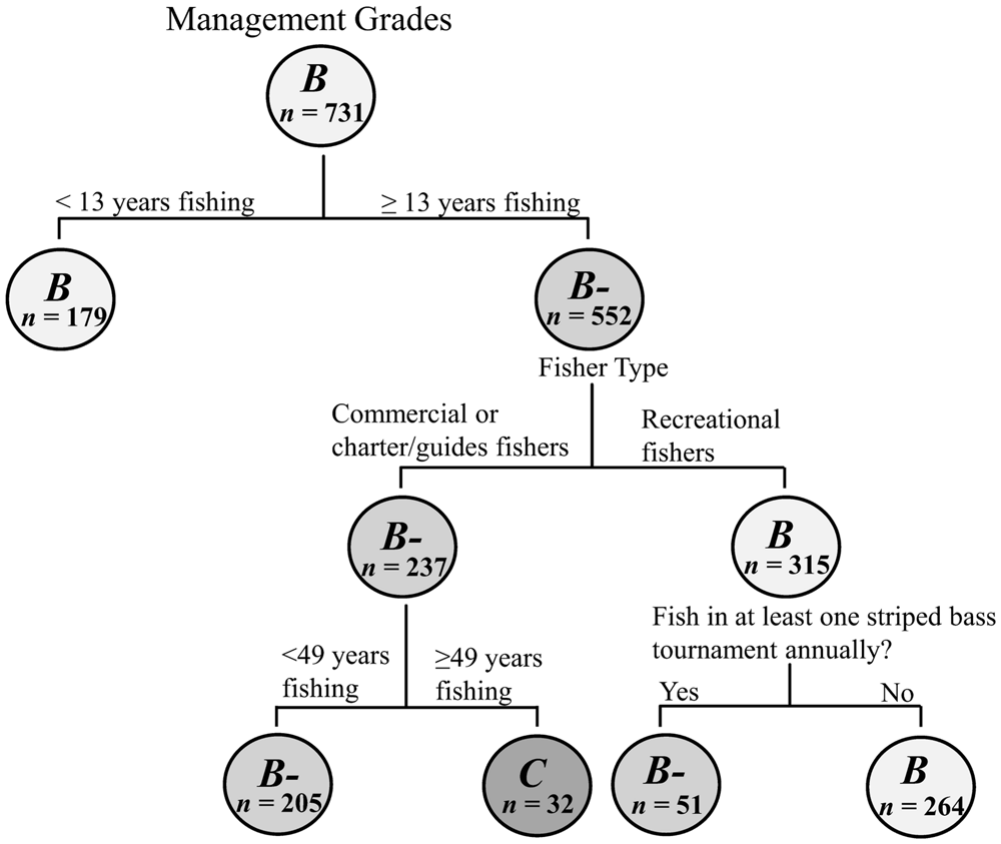
Classification tree of fishers’ perceptions of management. Letters in each bubble correspond to the median grade for each group, while numbers represent the sample size. Variables predict grades based on their relative placement on the tree, where the highest variable explains the maximum variation. All splits shown are significant at *P* < 0.05 and were predicted according to *LogWorth* values.

Participants were asked to grade their state’s current management of the striped bass fishery on a typical *A+* to *F* scale. Classification tree analysis revealed that “striped bass fishing experience” was the strongest predictor of angler management grade (Fig 1): those that have been fishing for fewer than 13 years assigned a median grade of a *B*, while those with 13 or more years of experience were slightly more critical and assigned a median score of a *B-*. For more experienced anglers, “fisher type” was the strongest explanatory variable. Commercial fishers and charter boat captains/guides were statistically non-distinct and gave management a *B-* grade, while recreational fishers assigned it a *B*. Commercial anglers and charter boat captains/guides could be further classified by fishing experience. Anglers with 49 years of experience or more had the lowest opinion of striped bass management with a median score of a *C*, compared with a median score of *B-* from those with less than 49 years. Lastly, recreational anglers’ degree of participation in tournaments was a predictor of their perceptions of the effectiveness of striped bass management efforts in their fishery: anglers that participated in a tournament were slightly less positive of management and assigned a median grade of a *B-*, compared to a *B* from the non-tournament anglers.

### Overall receptiveness and perceived effectiveness of regulations

**Fig 2:**
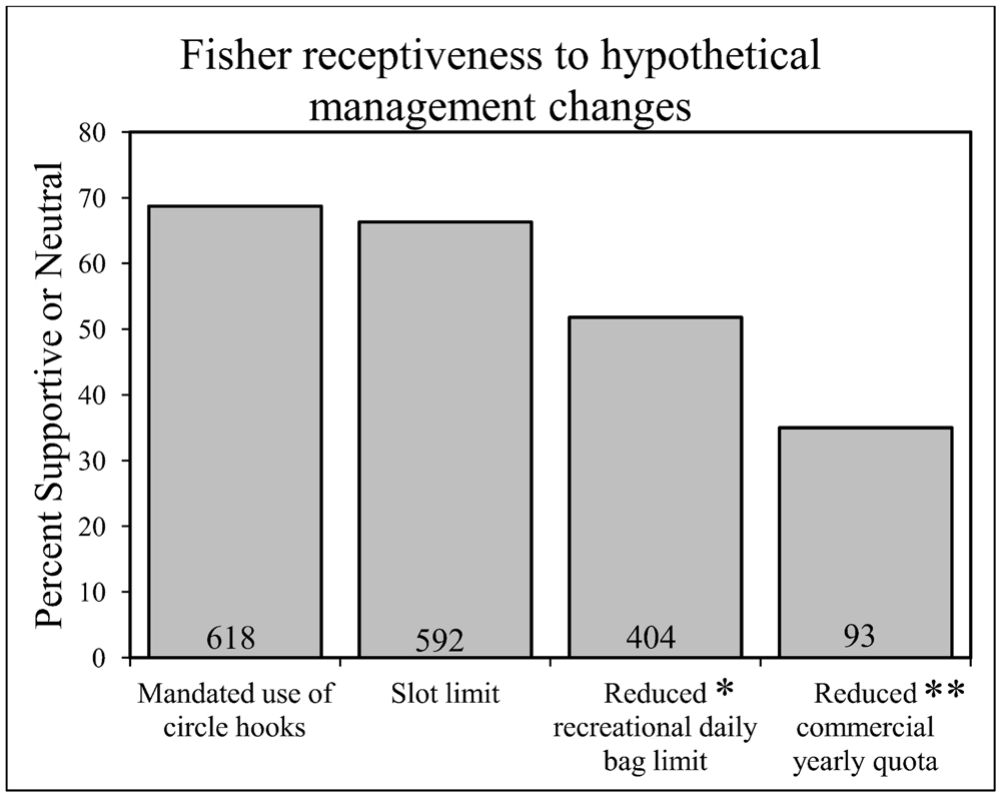
Percent of total response for participants that are supportive/neutral towards four management changes. Numbers in each bar represent the number of participants with supportive/neutral responses. *Reduced recreational daily bag limit includes responses from only recreational anglers. **Reduced commercial yearly quota includes responses from only commercial fishers.

Both recreational and commercial anglers were generally amenable to most of the different management strategies that were offered. The management alternatives with greatest support included mandating circle hook usage and implementing slot limit regulation changes, with 68% (*n* = 900) and 66% (*n* = 893) of participants selecting supportive/neutral options for each alternative, respectively. Opinions on the reduction of recreational bag limits were reasonably split down the middle (52% supportive/neutral, *n* = 780). Additionally, 35% (*n* = 266) of commercial anglers were supportive or indifferent towards a reduction in the commercial industry’s yearly quota (Fig 2).

**Fig 3:**
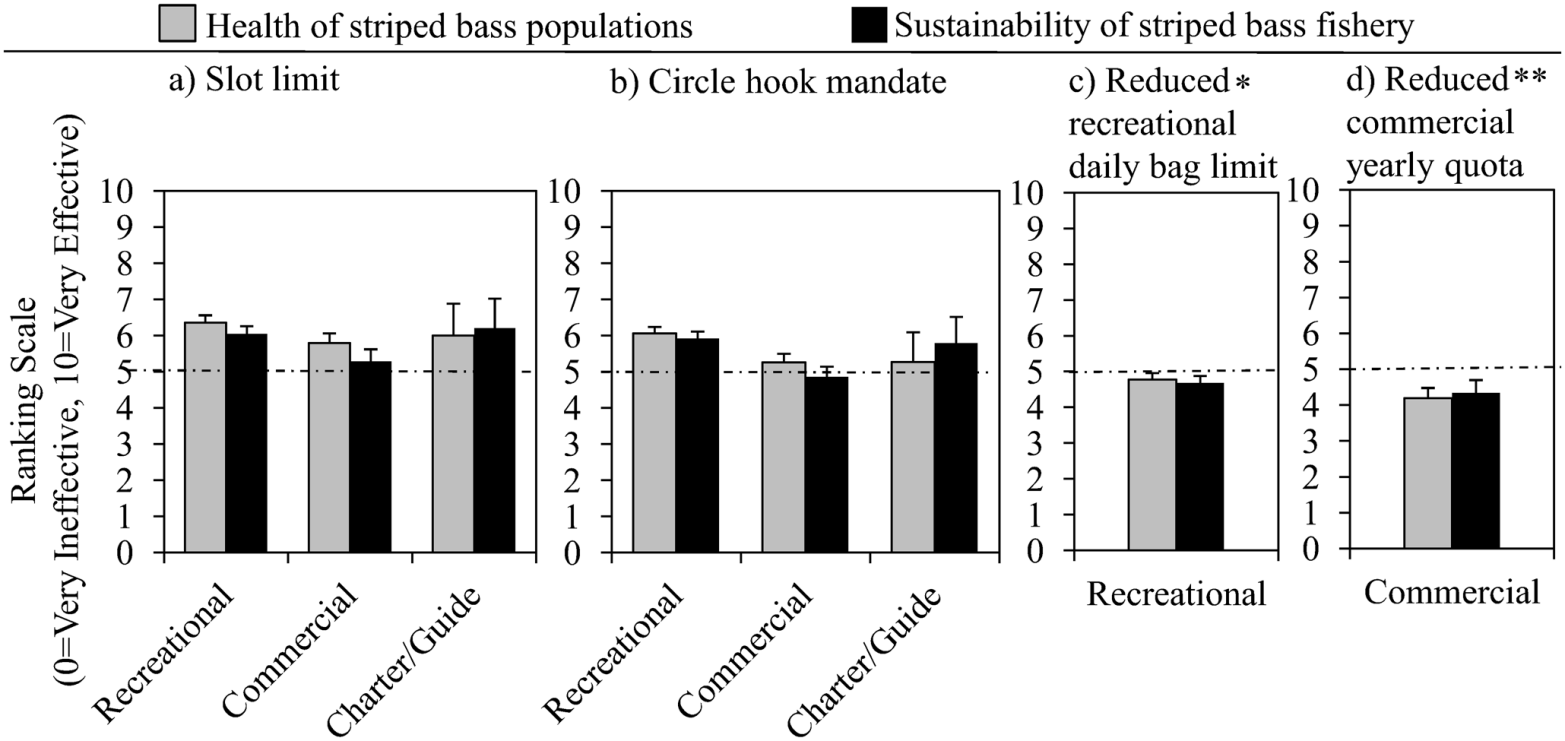
Effectiveness of hypothetical regulations. Mean ranking +1SE of the effectiveness of proposed regulations by “fisher type,” where a score of 10 correlates to maximum effectiveness. Proposed regulations are as follows: a) Slot limit, b) Circle hook mandate, c) Reduced recreational daily bag limit, d) Reduced commercial yearly quota. *Reduced recreational daily bag limit includes responses from only recreational anglers. **Reduced commercial yearly quota includes responses from only commercial fishers.

All stakeholder groups in our survey believe regulation changes will have similar impacts, respectively, on the health the fish population and fishery. Both recreational and commercial anglers perceive the implementation of a slot limit to be equally effective at promoting the health of striped bass populations and promoting the sustainability of the fishery (recreational; *P* = 0.1177, commercial; *P* = 0.3025, charter boat captains/guides; *P* = 0.9813, Fig 3a). Participants from both fisheries perceived the effectiveness of circle hooks to be equivalent for both categories as well (recreational; *P* = 0.8916, commercial; *P* = 0.3060, charter boat captains/guides; *P* = 0.3858, Fig 3b). Recreational anglers responded similarly to the effectiveness of a reduced recreational daily bag limit (*P* = 0.6816, Fig 3c), as did commercial anglers to the effectiveness of a reduced commercial yearly quota (*P* = 0.6058, Fig 3d).

### Implementing a Slot Limit

**Fig 4:**
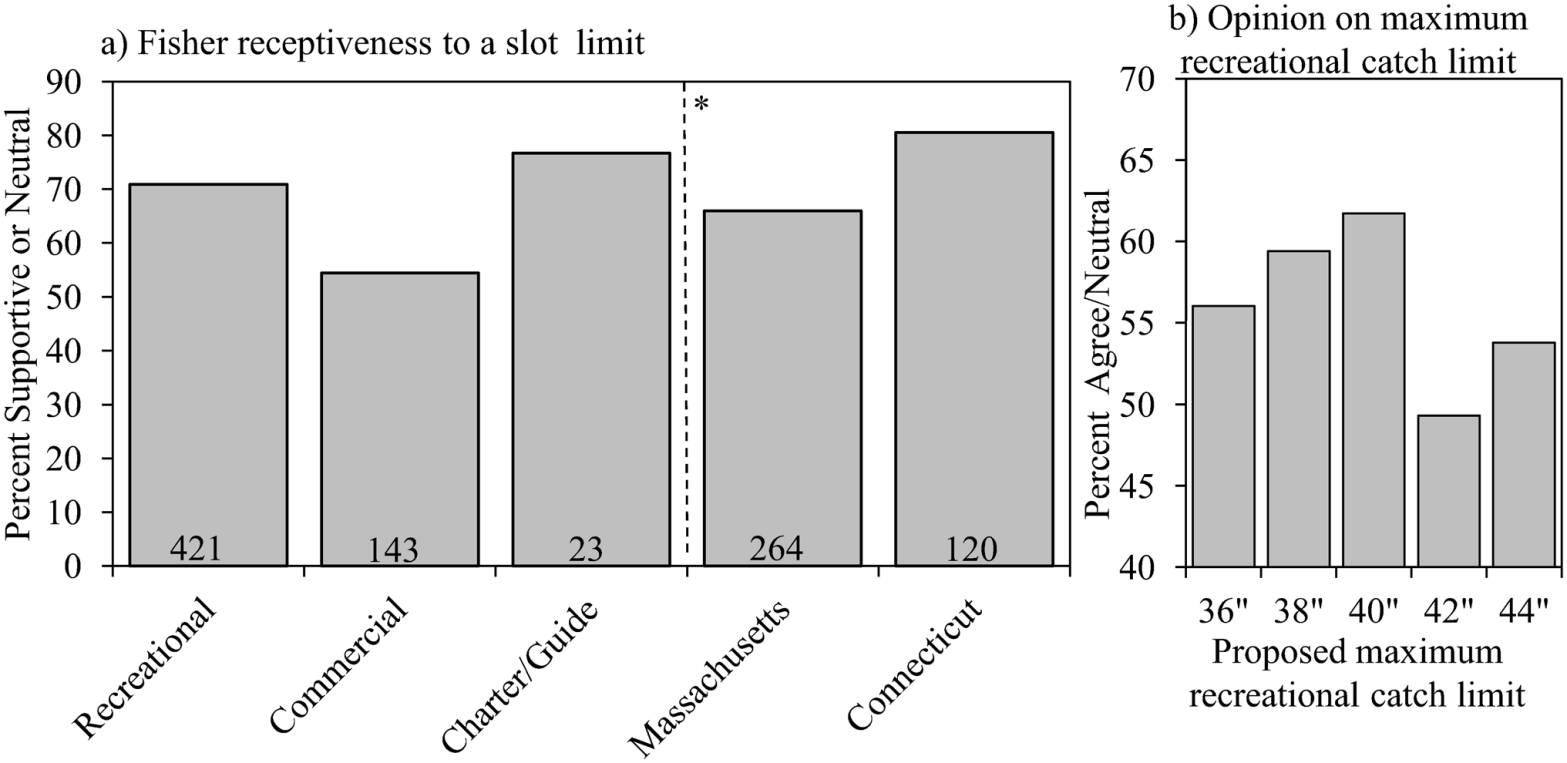
Slot limit analysis. a) Percent of total response for participants by “fisher type” and “state” that are supportive/neutral to the implementation of a slot limit. Numbers in each bar represent the number of participants with supportive/neutral responses. *Respondents did not include commercial anglers. b) Percent of total response for recreational anglers that agree with or are neutral towards a randomly assigned maximum allowable size for recreational striped bass harvest.

As a whole, recreational fishers were very supportive (71%; *n* = 594) of implementing a slot limit, as were charter boat captains/guides (77%; n = 30, *P* < 0.001, Fig 4a). Least supportive were the commercial anglers, but the majority (54%; *n* = 263) of these participants still selected supportive or neutral responses. When grouped by state, CT recreational anglers and charter boat captains/guides had a non-negative response rate of 81% (*n* = 149), and were more receptive than their MA analogues (66%; *n* = 400; *P* < 0.001, Fig 4a). While the following results are not statistically significant, analysis of randomly assigned upper size limits identified a slight trend of peak support at 40”, where the majority of participants displayed positive or neutral opinions (*P* = 0.18, Fig 4b). Support decreased slightly for shorter maximum-lengths, whereas there was a sharp decline for limits of 42” and 44”. Classification tree analysis generated only one strong predictor variable capable of explaining variation in support for a slot limit regulation change: “State.”

### Mandating Circle Hook Usage

**Fig 5:**
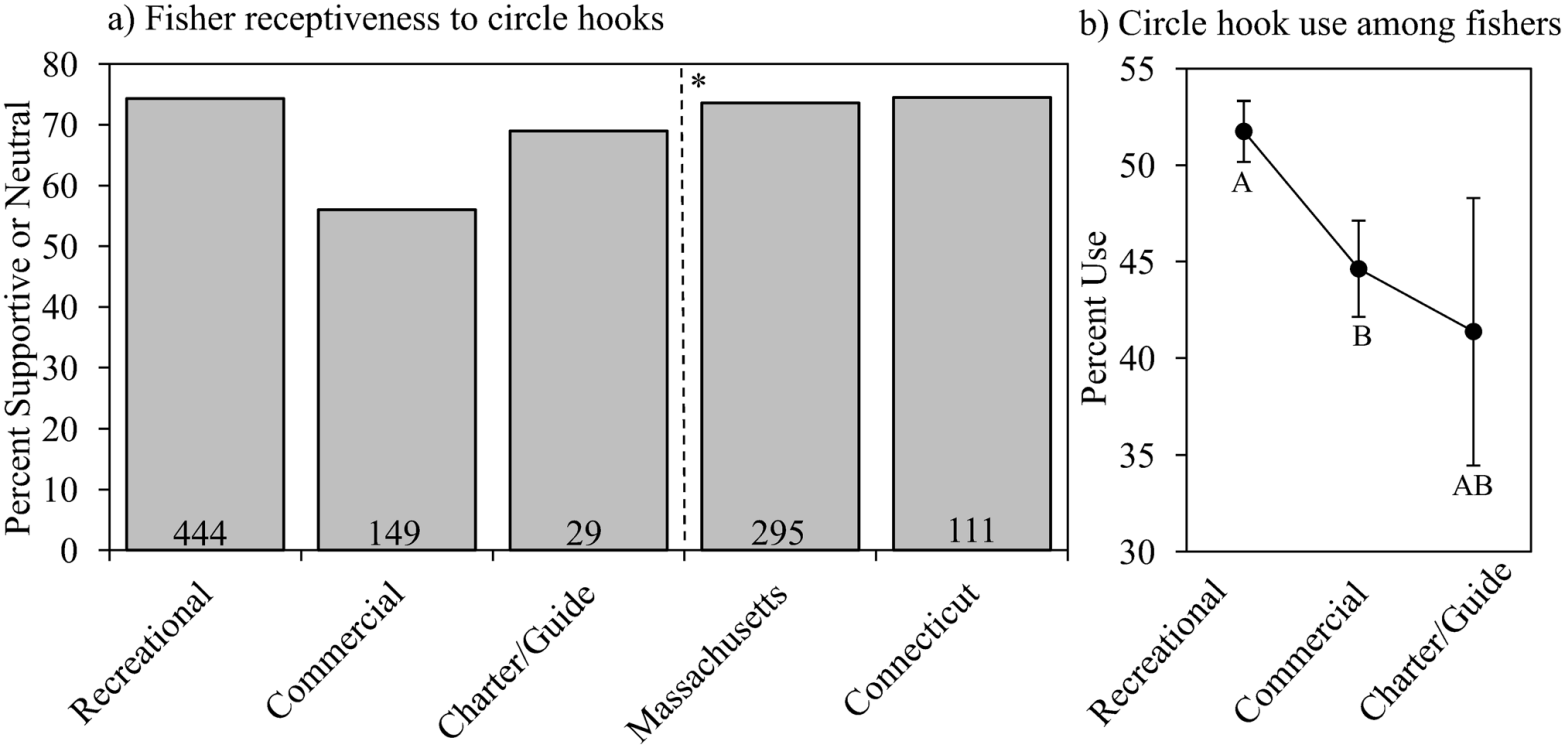
Circle hook analysis. a) Percent of total response for participants by “fisher type” and “state” that are supportive/neutral to mandated circle hook usage. Numbers in each bar represent the number of participants with supportive/neutral responses. *Respondents did not include commercial anglers. b) Mean ± 1SE of the percent of time participants use circle hooks when fishing for striped bass by “fisher type.” Letters below error bars are the results of a Tukey’s post-hoc test.

**Fig 6:**
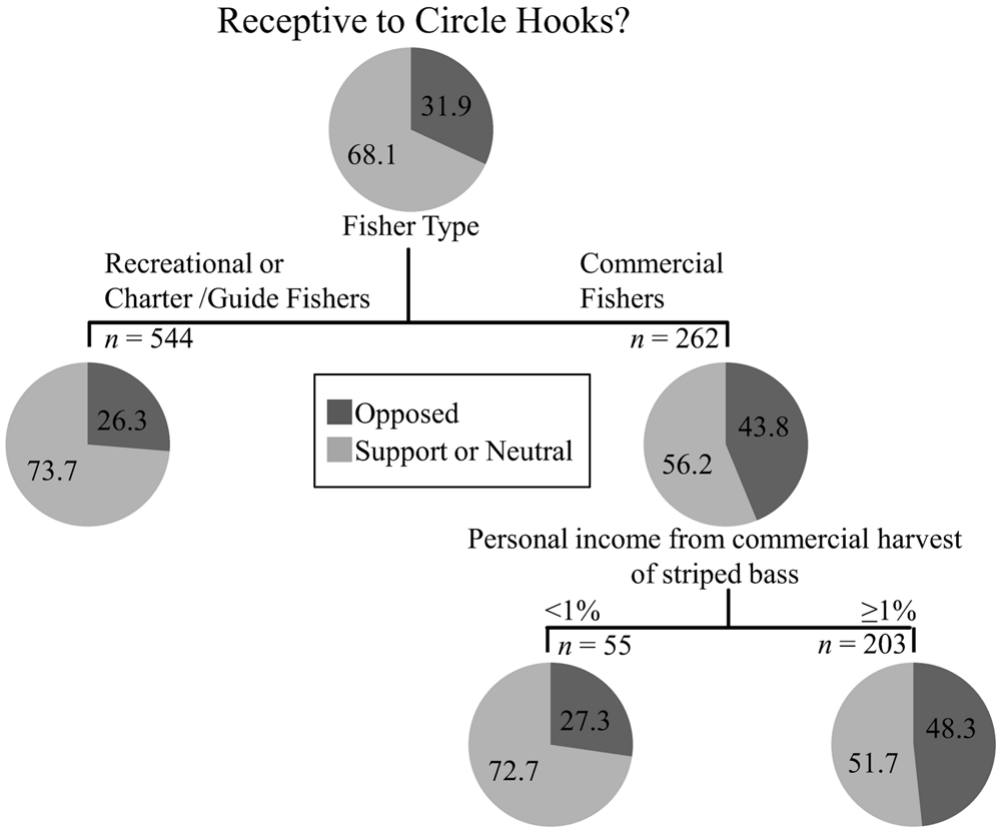
Classification tree of circle hook analysis. Variables predict support based on their relative placement on the tree, where the highest variable explains the maximum variation. All splits shown are significant at *P* < 0.05 and were predicted according to *LogWorth* values. Numbers in each bubble correspond to the percent response for each category.

Similar to their perception of implementing a slot limit, commercial anglers were indifferent or supportive of mandating circle hooks slightly more than half of the time (56%; *n* = 262). Recreational anglers were highly supportive with a 74% (*n* = 598) non-negative response rate. Charter boat captains/guides remained intermediary at 69% (*n* = 38). All fisher types were significantly different from one another (*P* < 0.001, Fig 5a). Perceptions of mandating circle hook usage among recreational anglers and charter boat captains/guides from each state were largely similar with non-negative response rates at 74% (*n* = 401) in MA and 75% (*n* = 149) in CT (*P* = 0.825, Fig 5a). “Fisher type” was a strong predictor of circle hook usage, as recreational anglers used circles hooks significantly more than commercial anglers (recreational anglers; 52%, commercial anglers; 45%, *P* = 0.0181, Fig 5b). There was a trend of slightly less circle hook usage by charter boat captains/guides (41%, Tukey’s post-hoc test, Fig 5b). Results of classification tree analysis produced two explanatory variables of participant receptiveness to mandating circle hook usage: “fisher type” and “percent personal income from the commercial harvest of striped bass” for commercial anglers (Fig 6). The former is the strongest predictor, as commercial fishers were supportive or neutral 56% percent of the time (*n* = 262). Recreational fishers and charter boat captains/guides were considered statistically non-distinct and, as a whole, displayed a 74% non-negative response rate (*n* = 636). Within commercial anglers, those that rely on striped bass harvest for 1% or more of their annual income were the most opposed to mandating circle hook usage, although roughly 52% of respondents were still supportive or neutral towards this regulation change (*n* = 203).

### Reduced Recreational Daily Bag Limit

**Fig 7:**
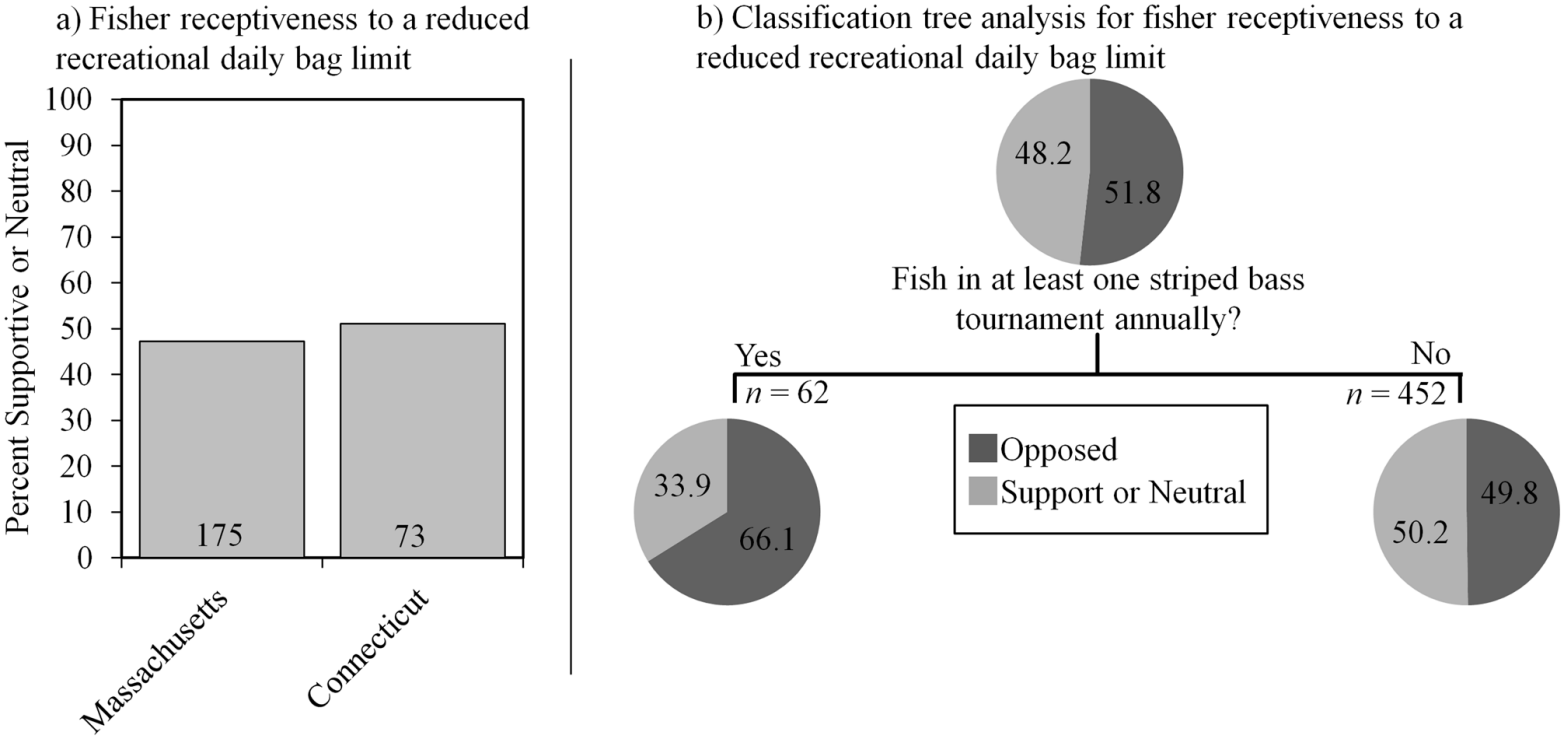
Reduction in recreational daily bag limit analysis. a) Percent of total response for participants by “state” that are supportive/neutral to reducing the recreational daily bag limit. Only recreational anglers were asked this question. Numbers in each bar represent the number of participants with supportive/neutral responses. b) Classification tree analysis depicting the percent of fishers who are supportive/neutral or opposed to reducing the recreational daily bag limit. Variables predict support based on their relative placement on the tree, where the highest variable explains the maximum variation. All splits shown are significant at *P* < 0.05 and were predicted according to *LogWorth* values. Numbers in each bubble correspond to the percent response for each category.

In MA, 47% (*n* = 371) of recreational anglers were in favor of or indifferent to reducing the recreational daily bag limit from two down to one fish per day. These results were not significantly different from CT, where 51% of recreational anglers were supportive or neutral (*n* = 143; *P* = 0.4303, Fig 7a). Classification tree analysis revealed that tournament participation was the strongest predictor of support for bag limit reductions. In particular, anglers that participate in tournaments were less supportive (34% non-negative response rate, *n* = 62, Fig 7b) than non-tournament anglers (50%, *n* = 452).

### Reduced Commercial Yearly Quota

**Fig 8:**
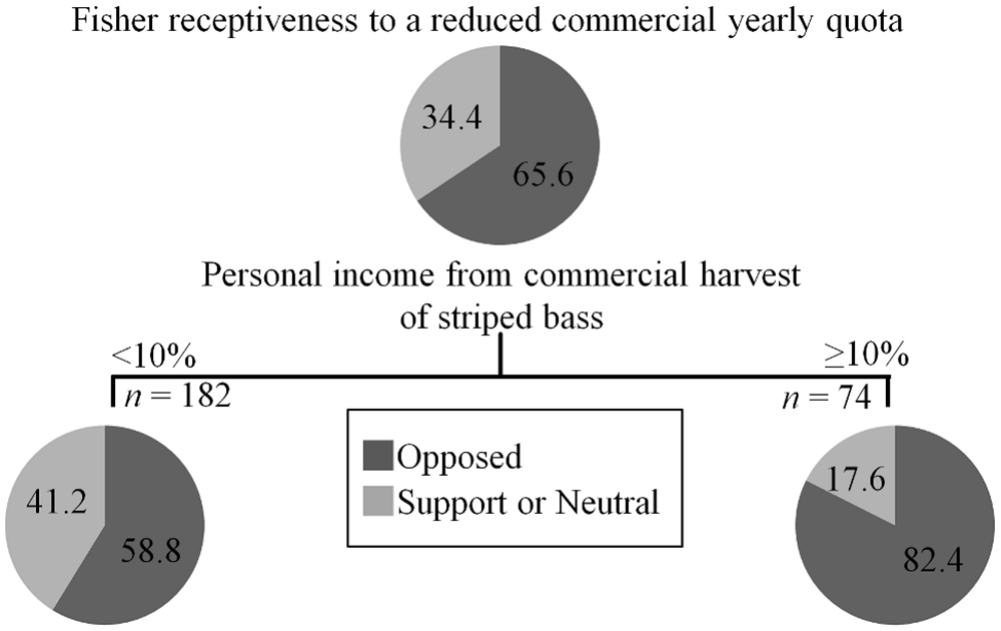
Classification tree analysis depicting the percent of fishers who are supportive/neutral or opposed to a reduction in the commercial yearly quota. Only commercial anglers were asked this question. Variables predict support based on their relative placement on the tree, where the highest variable explains the maximum variation. All splits shown are significant at *P* < 0.05 and were predicted according to *LogWorth* values. Numbers in each bubble correspond to the percent response for each category.

Analysis of a reduced commercial yearly quota was not possible by either “state” or “fisher type” since only commercial anglers were included and there is no commercial harvest in CT. Classification tree analysis revealed “percent personal income from the commercial harvest of striped bass” as the most powerful predictor of support (Fig 8). Anglers that derived less than 10% of their income from striped bass fisheries displayed a non-negative response rate of 41% (*n* = 182), versus 18% for their counterparts (*n* = 74).

## Discussion

Incorporating social dynamics into fisheries management is necessary for a holistic approach to ecosystem-based management [25]. Engaging stakeholders in the management process is also central to the development of effective governance structure [26–27] because it likely will increase fisher compliance to regulations [28]. For instance, understanding the perceptions of these stakeholders can help identify policy changes that anglers would be highly amenable to. Our survey revealed that New England striped bass fishers have positive perceptions of both mandating circle hook usage and implementing a slot limit regulation, the former of which has been proposed to benefit striped bass by reducing post-release mortality [29].

Fishers’ compliance and awareness of new regulations will likely mediate whether these regulations are successfully implemented. For instance, a study in Minnesota on the northern pike freshwater recreational fishery revealed low compliance and a lack of awareness of slot limit regulations, such that over 10% of fish harvested were of illegal sizes [30]. Fisher compliance to policy changes would in part depend on their perceptions of the efficacy of these proposed management policies. Furthermore, adopting policies that anglers are amenable to could reduce illegal activities and enhance their overall trust in fisheries management [31]. Considering that policy enforcement is dependent on limited federal and state budgets, a self-regulating system of compliant stakeholders could lead to more effective long-term management.

More experienced anglers comprised a large subset of our sample and held mixed attitudes towards management. Angler dissatisfaction with management may be typical among this group or could possibly be associated with historical striped bass population trends or with changes in policy. In addition to experience level, financial reliance on the commercial fishery seemingly influences the degree to which they are supportive of how striped bass is being managed. On the other hand, while recreational anglers may not be economically-dependent on the fishery, the cultural significance of the recreational fishery is substantial, as striped bass are one of the primary inshore fish species targeted in New England and are caught by tens of thousands of anglers annually. However, recreational anglers maintained generally positive viewpoints towards striped bass management and potential regulation changes.

While the effectiveness of either a slot limit or mandating circle hooks for sustaining striped bass populations involves scientific uncertainty, our work demonstrates that overall many fishers would be supportive of such management changes. Additionally, almost all fisher types in our survey, but particularly among recreational anglers, seem to support the implementation of a slot limit and mandating circle hooks, since they believe it will aid in both the proliferation of striped bass and the success of the fishery. These results suggest that participants perceive a strong connection between the health of the ecosystem and the striped bass fishery. Resource systems where the participants understand the connection among the ecosystem, fish populations and the fishery may enhance angler compliance with regulations [32]. Conversely, future assessments could use similar survey techniques to identify resource systems where there is a perceptional disconnect between the resource and industry. In these instances, education and outreach efforts would be aimed at minimizing gaps in understanding.

To elaborate on fisher perceptions of slot limit regulations, we asked participants to express viewpoints of randomly assigned maximum harvest lengths. Despite the absence of significant differences between proposed slot maximums, anglers seemed to identify 40” as their preferred limit. This potential threshold may reflect a tradeoff between reducing harvest of large female striped bass and fisher satisfaction. Specifically, maximum harvest lengths of 36” and 38” may result in the release of more fish than many anglers prefer. Meanwhile, the lack of support for higher limits may indicate that anglers believe that longer maximum catch sizes would not have significant, positive impacts on striped bass abundance. Future research should investigate why anglers are in favor or against specific optimal size minimums and maximums to better gauge potential compliance of alternate options within one regulation category. It is plausible that high compliance may occur at one maximum size limit that is well supported, but at another that is not, poaching may increase to a point such that the regulation’s costs are greater than its benefits. However, angler education could help push opinions in favor of scientifically sound regulations, thus increasing support and possibly compliance.

Limiting unnecessary mortality is a high management priority, especially for highly valuable game fish species where recreational anglers may release fish in an unsustainable manner [21]. From personal communication with both recreational and commercial anglers, many individuals already use circle hooks due to the perceived reduction in release mortality, which may be as high as 70% for striped bass [33]. This perception is in agreement with research on the use of circle hooks; they have been shown to reduce post-release mortality and injury for striped bass by 12.5% [29, 34]. Our results suggest that a policy mandating circle hook usage would be widely supported likely due to the perceived increases in striped bass survival post catch-and-release. Recreational fishers already use circle hooks more than half of the time while fishing for striped bass, and adopting this policy would likely shift circle hook usage closer to full compliance. While we are not advocating for or against this regulation (or any of the included for that matter), we simply highlight the potential sources of and reasoning behind angler perceptions of each management strategy.

There is considerable support for the implementation of a slot limit and mandating circle hooks, but support for other management alternatives such as a reduced recreational daily bag limit is lacking. Among other recreational regulations in our survey, this could potentially have the largest impact on fishing mortality, yet angler support is low in comparison. With a current two fish per day regulation, anglers are seemingly opposed to further decreases in harvest rates, which seems to be a consistent attitude across states. Most extreme among this participatory group was tournament anglers. The competitive nature of tournaments may influence why these anglers are less supportive, or perhaps tournament anglers are more dependent upon the recreational fishery. Targeted outreach initiatives and assessments could occur at tournaments to evaluate fisher behavioral responses to regulation changes and could potentially aim to mitigate social and cultural impacts (e.g., stakeholder conflict) of policy.

There was even less support for reducing the commercial quota, but still a third of commercial anglers were neutral or supportive of this change. This can be attributed to the relatively low financial reliance of striped bass anglers on the fishery for income, or perhaps signifies that many anglers perceive long-term benefits for striped bass populations, and hence the sustainability of the fishery, from a reduction in harvest levels. Our results suggest, however, that minimal reliance (≥10% of annual income) corresponds with largely reduced support for this regulation change. These commercial anglers are overwhelmingly against quota cuts and consequently should be included in the previously mentioned outreach initiatives targeting heavily impacted stakeholder groups. Making these results even more pertinent, recent restrictions limit commercial fishing to Mondays and Thursdays with a 15 fish per day bag limit. The public announcement of these regulation changes occurred two months after the release of our survey. Including this type of social analysis into management decisions could give managers insight into non-compliant stakeholder groups and may inform decisions among multiple regulation options.

To note, our results may be subject to response bias such that responses could be skewed towards experienced and specialized anglers. Responses were solicited using an email that specifically indicated that we were conducting a survey of striped bass anglers, potentially increasing the response rate in favor of anglers who place higher importance on striped bass or those with increased recreation specialization [35]. However, the comments that we received and the demographic information that we collected as part of the survey indicated broad representation of recreational and commercial striped bass anglers, and consequently suggests that this bias was likely modest and did not significantly influence the presented results. Furthermore, while data for ‘How many years have you been fishing for striped bass?’ is of a non-normal distribution, the results suggest that respondents span a breadth of fishing experience levels including a large number of extremely new anglers (<5 years fishing experience). Additionally, the monetary incentive placed on the completion of the survey likely reduced non-response bias. Disparate response rates from MA and CT anglers also suggests a higher level of interest among MA anglers, since our email correspondence specifically listed that we were conducting a survey of striped bass anglers.

Online surveys inherently exclude a portion of anglers without computer access or email addresses, potentially resulting in coverage error. Despite this bias, computer use is becoming universal, making it more efficient for researchers to utilize online-based surveys, while also providing them with representative sample responses. As an example, more than 70% of commercial anglers listed their email address in the database provided to us by MA DMF highlighting the near ubiquity of computer use in our sample population of anglers.

Results from this study must be conscientiously applied to other systems. For example, commercial striped bass anglers in our survey derive on average 10% of their personal income from the harvest of striped bass. It is not uncommon for commercial striped bass anglers to have occupations outside of fishing, thus potentially increasing the likelihood that they would support management changes in general. Additionally, the mode of the total household income for respondents is between $100,000 and $150,000 suggesting that our results may not be generalizable to other less financially stable fishing communities in other fisheries. As a whole, recreational anglers indicated that roughly half of total striped bass fishing effort is strictly shore-based and does not involve the use of a boat. As a shore-bound angler in New England, large bodied gamefish seldomly can be easily accessed. This may influence the perceptions of anglers due to a potentially larger proportional investment in striped bass fishing as compared to other geographic regions that may harbor a higher diversity of shore-based fishing options. Future assessments should aim to capture responses from a broader array of socioeconomic backgrounds and recreational settings in order to make generalizations across regions and fisheries.

Our study revealed that the perceptions and responses of key stakeholders to existing and proposed fishery regulations can be assessed with online surveys, which should aid decision making by managers. To select strategies that will garner higher relative compliance rates, management agencies could utilize similar survey techniques to assess stakeholder viewpoints prior to the implementation of a policy or the restructuring of existing regulations. To note, recent stock assessments have resulted in proposed new regulation requirements for coastal states [36], and will likely involve one or more of the regulations in this survey. Therefore, future assessments should examine potential differences between hypothetical and realized support for management changes to determine the degree to which surveys of fisher perceptions of management can be used effectively to guide management decision making. It is quite possible that responses will vary and will show decreased support after the enactment of a regulation.

While anglers within the striped bass fishery generally perceive management as adequate or better, perspectives differ by state and group membership. Differing perspectives may also be present within regulations, such as slot limit maximums, and could potentially influence compliance post-regulation implementation. Additionally, increased integration of fishing into an individual’s hobbies or livelihood, here in the form of tournament participation and financial reliance, seem to negatively influence the magnitude of their support. By identifying groups that are less receptive to proposed regulation changes, managers can develop strategies to minimize stakeholders’ financial losses or target outreach efforts at these groups to educate them on the benefits of a proposed management alternative. Ideally, this approach helps increase trust and compliance and thus, reduces conflict and illegal harvest. Used in conjunction with population dynamics and ecosystem-based modeling, data on fisher perceptions derived by surveys such as ours can be used to weigh the benefits and costs of each potential regulation alternative.
